# Evidence of Gender Violence Negative Impact on Health as a Lever to Change Adolescents’ Attitudes and Preferences towards Dominant Traditional Masculinities

**DOI:** 10.3390/ijerph18189610

**Published:** 2021-09-12

**Authors:** Maria Padrós Cuxart, Silvia Molina Roldán, Elena Gismero, Itxaso Tellado

**Affiliations:** 1 Department of Teaching and Learning and Educational Organization, University of Barcelona, Passeig de la Vall d’Hebron, 171, 08035 Barcelona, Spain; mariapadros@ub.edu; 2 Department of Pedagogy, Universitat Rovira i Virgili, Carretera de Valls, s/n, 43007 Tarragona, Spain; 3 Department of Psychology and UNINPSI, Comillas Pontifical University, C. Universidad Pontificia Comillas, 3-5, 28049 Madrid, Spain; egismero@comillas.edu; 4 Department of Pedagogy, University of Vic-Central University of Catalonia, c/Sagrada Família, 7, 08500 Vic, Spain; itxaso.tellado@uvic.cat

**Keywords:** toxic relationships, gender violence, adolescents, peer group, masculinities, health

## Abstract

Women and girls experience gender violence from a young age. Scientific research has presented evidence of the negative impact of toxic relationships and toxic stress on physical and psychological health. However, less is known on how this evidence can have a preventive effect. Knowing these impacts can be important for women and girls to decide the type of affective-sexual relationships they want to have, and even transform their attraction towards different types of masculinity. This study presents results from the MEMO4LOVE project. Researchers use mixed-methods approaches, including a questionnaire (*n* = 141) to study adolescents’ peer groups’ interactions that promoted healthy or toxic affective-sexual relationships, and five communicative focus groups with boys and girls to analyze how these interactions can be transformed by sharing scientific knowledge on the effects of violent relationships with adolescents. The results showed the impact of sharing with adolescents the evidence of the adverse effects of toxic relationships with violent masculinities on health. The peer group’s transformation occurred: non-violent boys gained self-confidence, and girls redirected or reinforced their attraction to non-violent boys. These results suggest the potential positive effects of knowing the impacts of toxic relationships on girls’ health.

## 1. Introduction

Toxic relationships due to gender-based violence impact women’s physical and psychological health. Increasingly younger women and girls are experiencing this reality. Due to the devastating effects of violence against women on women’s health, it is increasingly being considered a public health issue [1].

The WHO [2] estimates that about one-third of women worldwide have suffered physical or sexual intimate partner violence or non-partner sexual violence at some point of their lifetime, and that 27% of women between 15 and 49 years old who have been in a relationship have been victims of physical or sexual violence by their partner. Among adolescents, 24% of girls aged 15 to 19 who have been in a relationship have suffered physical or sexual violence from their partner, and 16% of young women between 15 to 24 years old experienced this violence in the past 12 months [3]. 

Toxic stress related to adverse psychosocial situations can have harmful effects on the brain and overall health. These situations include psychological trauma due to threatened death, serious injury, or sexual violence [4]. The stress system mediates the stress response and involves both the central nervous system and peripheral organs. Consequently, the excessive or inadequate activity of the stress system can lead to behavioral and somatic pathologies [5]. For instance, exposure to psychological stressors can lead to PTSD (post-traumatic stress disorder) expressed through cognitive and emotional negative alterations [4]. In addition, the impact of psychosocial stress exposure on brain functioning associates with an increased risk of dementia in later life [6]. Although there is little research that connects gender-based violence to toxic stress, there is evidence that toxic stress is a consequence of women’s exposure to gender violence that can affect women’s central nervous, endocrine, and immune systems [7]. The review conducted by Tsuyuki et al. [8] concluded a relationship between violence against women victimization, a physiological stress response, and immune dysfunction. This review also points out that violence occurring in childhood or adolescence can permanently alter the stress response, causing damage to the physiological system that sustains it. Overall, data on violence against women show that it can negatively affect women’s physical, mental, sexual, and reproductive health. It can lead to depression, post-traumatic stress and other anxiety disorders, sleep difficulties, eating disorders, and suicide attempts, as well as headaches, gastrointestinal disorders, and poor overall health [2]. The toxic stress model has been scarcely studied in adolescents. However, emerging research in this field supports the extension of this model in adolescence [9]. In this regard, it has been found that violent environments can be toxic for adolescents and create health risks [10]. 

In Spain, where the present study was conducted, 32% of women aged 16 or older suffered some type of physical, sexual, or psychological violence from an intimate partner or ex-partner at some point in their life, and 11% experienced it in the past 12 months [11]. Furthermore, 70% of these women reported psychological consequences of these episodes of violence and 24% consumed substances such as drugs or alcohol. In addition, women suffering some intimate partner violence at some point in their life more frequently need health assistance (including health emergency assistance and psychological assistance). They have up to five times more risk of having suicidal thoughts than women who have not suffered such violence, which shows the lasting impact of violence on the health status of women through time. Young women aged 16–24 are an especially vulnerable collective, as they suffer sexual violence (11%), sexual harassment (60%), and stalking (26%) twice more than women aged 25 and older [11]. 

In this regard, the research found that intimate partner violence victimization in adolescents impacts higher depressive symptoms, poorer physical health status, and higher levels of health care utilization [12]. In addition, toxic affective-sexual relationships—that is, violent relationships which in many cases include sexual-affective violence, mistreatment, contempt, and verbal or non-verbal attacks, often leading to the acceptance of humiliations and mistreatment—influence the initiation and persistence of substance use and the onset of mental health disorders [13]. Although ongoing exposure to violence has a more considerable impact on health status than more distant exposure, both negatively affect health outcomes [12]. Data from Spain also shows that young women (aged 16–24) who suffered sexual violence talked about it more frequently than adult women. In addition, they more often shared these experiences with friends [11], suggesting the peer group’s important role in tackling these situations. The peer group is the context where romantic relationships start during adolescence [14], however, it can also be the context in which coerced relationships occur. This event can negatively affect girls’ psychosocial wellbeing, for instance, when the peer group pressures them to start a relationship [15], related to later relational victimization [16]. Previous research has found evidence of peer group pressure on girls to start a relationship [17] and of the mirage of upward mobility in the Spanish context. It consists of girls’ mistaken perception of having an intimate relationship with boys responding to dominant traditional masculinity and raising their attractiveness and status. At the same time, the contrary occurs, and the girls’ status and attractiveness decrease. It has been identified as one of the causes of gender violence [18].

Research has found that one main factor that intervenes in preventing or promoting violence against women is the masculinity model embodied by men and boys, but also the patterns of women’s and girls’ election and attraction towards different masculinities. The media significantly influences this election and interest (cinema, TV series, video clips, youth literature, etc.). Media tend to present male models who are sexist and violent as attractive, voiding of attraction egalitarian boys and men [19,20,21]. Furthermore, media creates an image of masculinity, eroticism, and affective relationships linked to violence. The terms “toxic masculinity” and “positive masculinity” have been used to refer, respectively, to hegemonic masculinities characterized by aggressiveness and domination on the one hand, and on the other hand, healthy masculine identities that are supportive of gender equality [22]. The latter would prevent gender violence and be conducive to healthy relationships and the psychosocial and physical well-being of boys and girls. In this regard, the role of boys in preventing adolescent dating violence has been emphasized in community-based intervention programs to improve adolescents’ health and wellbeing [23]. Other studies have differentiated between three different types of masculinity to understand the role of men in gender violence: dominant traditional masculinities (DTM), oppressed traditional masculinities (OTM), and new alternative masculinities (NAM) [24,25,26,27]. DTM represent inegalitarian, dominant, and violent attitudes of men that can lead to gender violence, but that at the same time are associated with attractiveness in the dominant coercive discourse socially transmitted. OTM are egalitarian and non-violent boys and men who, also due to the dominant coercive discourse, are seen as good friends but unattractive as intimate partners. Therefore, they are not seen as an alternative to DTM, and do not contribute to overcoming gender violence. Finally, NAM are non-violent and egalitarian boys and men who show self-confidence, actively positioning themselves against gender violence. They are attractive to girls and women, challenging the dominant coercive discourse and therefore being an alternative to overcome gender violence and to promote healthy relationships and emotional and physical wellbeing [28]. 

Preventing gender violence among youth is an increasing concern in public health. Making scientific knowledge about the causes and impacts of gender violence available to adolescents can be an instrument to help them make informed decisions on the type of affective-sexual relationships they want to have. This study hypothesizes that knowing the negative impact violent masculinities and toxic relationships have on health can help adolescents take such decisions. In addition, adolescents may even transform their attraction to different types of masculinity, preventing these toxic relationships from occurring, and creating a potential positive long-life impact on their health.

This study aims to deepen how attraction to violent masculinities can contribute to toxic relationships in adolescents’ peer groups, and how it can be transformed when youth are informed of the adverse effects of such relationships on their health. The results show that when adolescent groups are aware and knowledgeable about the negative impacts on health from toxic relationships with violent masculinities, they change attitudes and/or preferences regarding intimate relationships. In addition, adolescents counteract social pressure towards non-egalitarian and potentially unhealthy relationships: non-violent boys gained self-confidence and girls redirected or reinforced their attraction to non-violent boys. These results suggest the potential positive effects of knowing the impacts of toxic relationships on girls’ health.

## 2. Materials and Methods

### 2.1. Research Design

This study is part of the larger research project “MEMO4LOVE. Social interactions and dialogues that transform memories and promote sexual-affective relationships free of violence from secondary education schools” (2016–2020), funded by the Spanish Government. The project’s main objectives were to identify interactions and dialogues among adolescents that promote learning attraction to violence or non-violence in affective-sexual relationships and examine the impact of preventive socialization of gender violence. MEMO4LOVE was conducted in two stages. The first stage consisted of an analysis of the interactions that occur in the adolescents’ peer group, how they reflect an attraction to violence or non-violence and its relation to gender violence. A sample of 141 adolescents (84 girls and 57 boys), most of them aged 14 and 15 years old from three different secondary schools (two public and one private) in Sevilla (Spain) participated in this stage. The second stage consisted of implementing an evidence-based program of seven sessions on preventive socialization of gender violence with adolescents aged 15 and 16 in three secondary schools (two public and one semi-private) in Barcelona (Spain) and analyze its impact [29]. The interventions were facilitated by researchers of the project and consisted of lecturing and establishing a dialogue within the class group about relevant topics related to gender-based violence, such as the social nature of attraction, the coercive dominant discourse, peer group solidarity, consent, and false assumptions on gender violence. We will focus on two interventions: the effects of toxic relationships on physical and psychological health (intervention 4) and the different types of masculinities and their role in gender violence or their prevention (intervention 5). 

The present study was conducted as an exploratory research with two objectives: (1) to analyze how attraction to violent masculinities showed in the context of the peer group can influence toxic relationships among adolescents, and (2) to analyze the preventive impact of knowing the harmful effects of toxic relationships and violent masculinities on health. 

A mixed-methods design comprising quantitative and qualitative data was conducted (see Section 2.2), following the communicative methodology of research [30]. This methodology aims to go beyond the description or understanding of reality to unveil the processes that can transform and overcome inequality, injustice, or violence. In this study, the communicative methodology was helpful to understand the processes that can transform adolescents’ preferences and relationships into non-violent, more egalitarian, and healthier, and was particularly relevant in the dialogues held in the qualitative part of the study. For this purpose, an egalitarian dialogue was established between researchers and the end-users of research, the adolescents, in which researchers shared their scientific knowledge on the topic of study, the adolescents shared their experiences, and new knowledge was created intersubjectively from the dialogue.

### 2.2. Data Collection Instruments

#### 2.2.1. Interactions Questionnaire

Within the first stage of the MEMO4LOVE project, a questionnaire created in the framework of the project was used to gather information about interactions in the adolescents’ peer group that reflect an attraction to violence or non-violence and its relation to gender violence. Five questions of the questionnaire were analyzed to respond to the first objective of the present study—to analyze how attraction to violent masculinities showed in the context of the peer group can influence toxic relationships among adolescents. These questions inquired how the peer group talked about each type of masculinity (DTM, OTM, and NAM), peer group pressure on girls to start a relationship and the existence of a mirage of upward mobility, which are examples of toxic relationships in adolescence. The responses to these questions help us understand to what extent adolescents are knowledgeable of these examples of toxic relationships, and how they relate to the group’s attraction to each type of masculinity.

#### 2.2.2. Focus Groups

In the second stage, a series of focus groups were conducted during and after the intervention program to study its impact. To respond to the second objective of our study—to analyze the preventive impact of knowing the harmful effects of toxic relationships and violent masculinities on health—the focus groups that were conducted at the end of the intervention program in each high school were analyzed. Three focus groups were conducted in school 1 (in three different class groups), and one focus group was conducted in school 2 and school 3 (a total of 5 focus groups). Following the communicative methodology of research, these focus groups help us understand the perceived impacts of the interventions program and which were the program sessions that were more significant for them. All focus groups were mixed (both girls and boys participated) and were conducted by researchers on the high schools’ premises. All focus groups were audio-recorded and transcribed verbatim to facilitate the subsequent analysis.

#### 2.2.3. Feedback Questionnaire

During the intervention program, a feedback questionnaire was used after each session for researchers to collect the adolescents’ impressions of each session of the intervention program. For this study, responses to the feedback questionnaire were analyzed, particularly the question that referred to how helpful the session was to reflect on their feelings and relationships (from “not useful at all” to “totally useful”). This quantitative and ongoing data collection was contrasted with the qualitative data of the focus groups collected at the end of the intervention program to triangulate the data and enhance the reliability of the results on the impact of the intervention program.

### 2.3. Data Analysis

#### 2.3.1. Quantitative Data Analysis

Quantitative analysis was conducted for the two questionnaires: the interactions questionnaire and the feedback questionnaire. For the interactions questionnaire, crosstabs were obtained, and descriptive statistics were used to analyze differences between peer group talk about each type of masculinity depending on the identified presence of peer group pressure, on the one hand, and the mirage of upward mobility, on the other hand. Responses regarding peer group talk about each type of masculinity were grouped based on scientific criteria. The typologies “Group talk showing acceptance and attraction” and “Group talk showing rejection and unattraction” were created. As adolescents could select more than one response for these questions, frequencies and percentages have been calculated based on the number of responses, and not on the number of participants. For the feedback questionnaire, descriptive statistics were used to analyze the data and compare the impact of the different sessions.

#### 2.3.2. Qualitative Data Analysis

Thematic analysis was conducted for the focus groups in order to identify and analyze patterns of shared meaning relevant to the research objective. A system of three categories was created following a deductive-inductive process, considering previous knowledge on the topic and the themes that emerged in the discussions with the adolescents. The categories are described in Table 1.

### 2.4. Ethics

The intervention program and the data collection at the project first and second stages followed all ethical standards for research involving human participants included in the Declaration of Helsinki [31] and Horizon 2020 (European Commission). At the beginning of each stage, the high schools’ boards were first informed of the project objective and all the activities that entailed their participation. Subsequently, written informed consents were obtained from the adolescents’ parents or legal guardians, and adolescents themselves were informed of the study’s objective and their right to participate or withdraw at any moment freely, as well as the anonymity of the data collected. Researchers were always available to respond and clarify any questions or doubts the participants may have to ensure their free and informed participation. The Ethics Committee of the Andalusia Government fully approved the study.

## 3. Results

### 3.1. Attraction towards Violent Masculinities in the Peer Group Context Can Have an Impact on Promoting That Girls Engage in Toxic Relationships

To respond to the first objective of our study, in this first section of the results, we analyze interaction patterns in the adolescents’ peer group regarding attraction to different masculinity models that can influence the risk for girls to engage in toxic relationships: on the one hand, to engage in relationships as a result of group pressure and, on the other hand, to engage in a relationship as a result of the mirage of upward mobility.

The interactions’ questionnaire analysis shows that attraction towards dominant traditional masculinities in the peer group—expressed in the way the group talks about this type of masculinity—is related to a greater probability of girls being exposed to peer group pressure to engage in a relationship and to the mirage of upward mobility.

Table 2 and Table 3 show the relationship between how the adolescents’ peer group talk is (showing acceptance and attraction or rejection and unattraction) about each type of masculinity (DTM, OTM, and NAM), and the existence of peer group pressure on girls to start a relationship (in Table 2) or the existence of the mirage of upward mobility (in Table 3).

Regarding peer group pressure, data shows that the peer group talks of boys representing each type of masculinity is related to the acknowledgement of peer group pressure towards girls to engage in a relationship. For youth who know about peer group pressure towards girls to engage in a relationship, the most frequent ways their peer group talk about DTM is with acceptance and attraction (66% of the responses are in this direction). In comparison, only the 16% of the responses reflect rejection and unattraction. For youth who do not know about peer group pressure towards girls to engage in a relationship, peer group talk about DTM shows a similar acceptance and attraction (63% of the responses), but higher rejection and unattraction (26%) (see Table 2).

In the case of OTM, for youth who know about peer group pressure towards girls to engage in a relationship, talk in their peer group about these boys include acceptance and attraction (44% of the responses) and rejection and unattraction (42%) similarly. However, the youth who do not know about peer group pressure towards girls to engage in a relationship has a higher percentage of responses showing acceptance and attraction (54%) and a lower percentage of responses of rejection and unattraction (31%) (see Table 2).

In the case of NAM, little difference can be observed between the way the group talks about them depending on the knowledge of cases of peer group pressure towards girls. Of those that do know such cases, 76% of responses included group talk about these boys showing acceptance and attraction and 9% showing rejection and unattraction. In contrast, the percentages for those who did not know such cases were 78% and 6%, respectively (see Table 2).

Therefore, when youth do not know of cases of peer group pressure towards girls, the group more frequently talk about DTM boys emptying them of attractiveness and talk about OTM with respect and interest. In contrast, when youth in the peer group know of cases of peer group pressure towards girls, verbal interactions are more frequently characterized by attractiveness towards DTM boys and disinterest towards OTM boys. Thus, globally—and especially comparing group talk regarding DTM and OTM boys—data indicates that verbal interactions that show attraction towards those representing a model of masculinity characterized by disrespect and violence and by disrespect towards those who represent fairness and kindness can be a risk factor for the existence of cases of peer group pressure towards girls. In contrast, attitudes of respect towards those representing good values and lack of attraction or interest towards those who represent violent values could have a protective effect.

Similarly, regarding the mirage of upward mobility, the interactions in the peer group regarding the different models of masculinity seem to be related to the probability of the existence of the mirage of upward mobility. When youth do know cases of the mirage of upward mobility, they more frequently talk about DTM boys showing acceptance and attraction (67% of the responses) and less frequently showing rejection and unattraction (15%). When youth do not know the mirage of upward mobility cases, the number of responses showing acceptance and attraction towards DTM boys reduces (55%). The number of responses showing rejection and unattraction towards these boys increases (30%) (see Table 3).

Regarding peer group talk about OTM boys, for youth who know the mirage of upward mobility, there is little difference between responses showing acceptance and attraction towards these boys (41%) and those showing rejection and unattraction (43%). The difference is more significant for youth who do not know the mirage of upward mobility cases: 58% of the responses show acceptance and attraction towards OTM boys, while 25% show rejection and unattraction (see Table 3).

Finally, looking at peer group talk about NAM boys, when youth know the mirage of upward mobility cases, most responses show acceptance and attraction (68%), and few answers show rejection and unattraction towards these boys (13%). When youth do not know the mirage of upward mobility cases, the percentages are similar, although slightly higher for responses showing acceptance and attraction (73%) and marginally lower for answers showing rejection and unattraction (13%) (see Table 3).

Therefore, peer group talk giving attractiveness to the boys showing a non-egalitarian and potentially violent model of masculinity may be a risk factor for the existence of cases of the mirage of upward mobility. In contrast, an upbeat talk about masculinity models showing egalitarian and non-violent values could be a protective factor.

### 3.2. When Sharing the Evidence of the Negative Impacts That Toxic Relationships Have on Health with the Group, Transformations in the Peer Group Occurred: Non-Violent Boys Gained Self-Confidence, and Girls Redirected or Reinforced Their Attraction to Non-Violent Boys

To respond to the second objective of this study, we explore the impact of gaining scientific knowledge on gender violence. We wished to discover the effect that learning about its causes and consequences, especially its effects on health, has on the prevention of engaging in violent relationships, either as attitudes or preferences that may lead to this type of relationship change, or attitudes or preferences that prevent violence are reinforced.

#### 3.2.1. Perceived Impact of Known Evidence of the Negative Consequences of Toxic Relationships on Health and the Role of Masculinities

In the focus groups with adolescents conducted at the end of the intervention program and in the feedback questionnaire they filled in after each session, girls and boys were asked about which intervention sessions were more important or had a more significant impact on them. Most of the youth who gave their opinion in the focus groups highlighted the intervention on toxic relationships’ effects on health. They explained that it was a novel topic for them, of which they had never heard before. They were impressed by the multiple and devastating effects of violent intimate relationships on psychological health and physical health. Both boys and girls agreed on the importance of that session for them to learn about the pervasive effects of toxic relationships, as can be seen in the following excerpt of a focus group:

Researcher: And of all the interventions we have made, of the topics we have discussed, which one has impacted you the most?

Girl 2: The one about how toxic relationships affect people, that thing about neurons and those things … I didn’t think it would affect that way, I was freaking out a bit.

Boy 1: Yes, I thought it affected psychologically, not your health.

(…)

Girl 1: It impacted us a lot because we were not aware of this knowledge.

Girl 2: Sure, they don’t tell you, if you have a toxic relationship, besides from the fact that someone can hit you and hurt you physically, they can also do it psychologically and health-wise. So… we didn’t know about that. I think that, for everyone, has been the one that has had the most impact. (FG school 1)

Although the session on the health consequences was the most impactful, the students also mentioned the intervention on the different types of masculinity and their role in gender violence in the focus groups. In this case, a girl mentioned both interventions together, referring to the consequences they learned violent relationships can have.

Girl 1: The one of good boy and bad boy [session about types of masculinity] … because you know what will happen, the consequences. The session about toxic relationships also because you know that if you get sick you get depressed and everything. And in terms of health, it is your health, and you can die too, so … (FG school 2)

**Figure 1:**
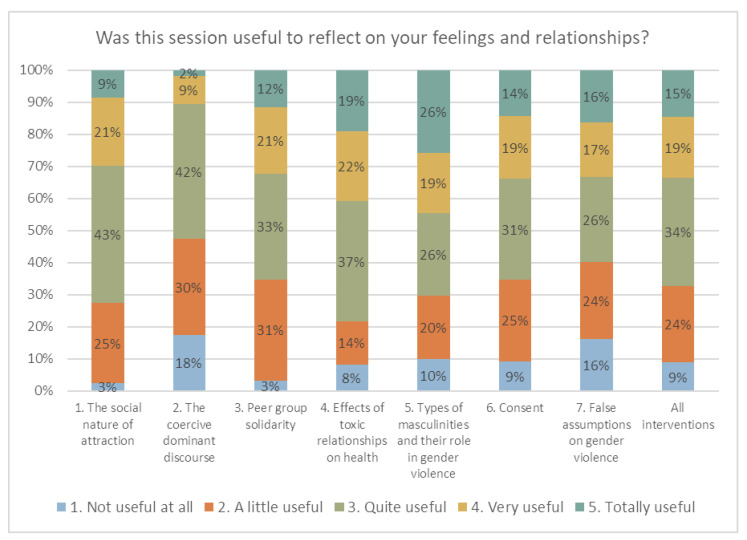
Utility perceived by adolescents of the different interventions.

These perceptions shown in the focus groups coincide with the feedback provided by the adolescents in the feedback questionnaire we asked them to fill in after each session. When we asked them whether the session was useful for them to reflect on their feelings and relationships, the session that had the greatest percentage of responses including “quite useful”, “very useful”, and “totally useful” was intervention 4, about the effects of toxic relationships on health (78%). Intervention 5, about the different types of masculinities and their role in gender violence, was the session with the most significant percentage of “totally useful responses” (26%). (see Figure 1)

Although the new information about the health impact of violent relationships surprised them due to its novelty, the students recall as highly useful the information about the different types of masculinities, with a particular focus on the role of violent masculinities in gender violence and that of new alternative masculinities in preventing it. In this regard, when calculating the average score for each session, we can see that the sessions that the youth perceived to be most valuable were intervention 4 and intervention 5, as these two sessions surpassed the average score of all sessions with almost the same punctuation (3.30 and 3.31, respectively) (see Table 4).

#### 3.2.2. Non-Violent Boys Gain Self-Confidence in the Peer Group

As expressed in the focus groups, the main consequence for boys of participating in the project interventions was that non-violent boys gained self-confidence within their peer group. In some cases, boys said that the interventions did not cause a change in their thoughts as they already thought that way—that is, they already held respectful non-violent attitudes—but the interventions helped them “to be more confident” or “to clarify their ideas a little more” (FG school 1). 

In addition, they reinforced their non-violent attitudes, started to see “cool” boys with violent or non-egalitarian attitudes as less attractive, and gained arguments to respond to peer group pressure to adopt behaviors typical of the dominant traditional masculinities. One of the participant boys even thanked the researchers after one of the sessions for sharing the scientific evidence on gender violence and the impacts of the different types of masculinities, as it made him more confident in his attitudes and behaviors. The following excerpt of one of the focus groups exemplifies this impact on boys:

Boy 2: Well, I’ve been told more than once, “hey, hook up with this person”, just because, and I say: “hey, I don’t know that person, I’ve known her shortly and she is not the type of person I like”, and they said: “no, do it, that way you look cool”, and I say: “Well, no, I don’t want to look cool, I want to look like someone normal, I don’t want to look cool either saying ‘I hooked up with this girl just because’, because for me that’s not being cool. Because I have had acquaintances who say “I have hooked up so many times or I have fucked so many times”, and I say: “look, that won’t make you better than me”. And well, I always tell them this argument because I say: “even if you do this more than others, you will not be better than someone else, you may be even worse.” I always say this statement and they say: “OK, I understand you”, and I say: “OK, then don’t repeat it, because if that happened to you, I wouldn’t tell you.” And with that argument and a little more talking with them, they already understand it, but then there are others who go on and on and on … and I say “nope” “why?” “Because I say so and that’s it.”

Researcher: And how has the project helped you on this issue?

Boy 2: To become aware of what I really want and not what others want for me.

Researcher: And besides from becoming aware, how has it helped you to respond to these situations?

Boy 2: Being honest and dismissing the opinion of others, not being at that level of today’s society, that the more flings you have and the more hook ups you have, the better person you are and way cooler, well no. The fact of practically not wanting to be like that average who says to prefer that, and well, mainly, that. (FG school 1)

#### 3.2.3. Girls Redirect Their Attraction to Non-Violent Boys

In the case of girls, the main impact recorded was that after learning about the consequences of violent intimate relationships and the types of masculinities associated with gender violence or its prevention, they started to redirect, or reinforce, their attraction to non-violent boys. Some participant girls acknowledged feeling attraction for the “bad boys”, but the project interventions made them think about whether this kind of boy was what they wanted. They had in mind what a relationship with such boys could bring to them, and comparing with “good boys”, as this girl explained:

Girl 1: Let’s see, it is true that the bad guy is the one who attracts the most and all that, but then after these talks you realize: “feeling attracted to that person doesn’t give me anything” and then you go looking for the good things of other people and you say: “I am more attracted to the one who is not bad than the bad one.” (FG school 1)

Importantly, as this girl explained, transformations in attraction occurred in two directions simultaneously: at the same time that they started to dislike “bad boys”, they began to pay attention and like “good boys”. Other girls’ interventions reflected in the same direction:

Girl 2: With the interventions, we have realized how bad others [“violent boys”] are because …

Girl 3: … you appreciate the others [“non-violent boys”] more. (FG school 3)

This change in their preferences transforms their ideal of relationship, which moves away from violent attitudes and approaches more egalitarian ones. Girls associate “bad boys” with toxic relationships, which they learn to reject due to the consequences they entail:

Researcher: What relationships do you dream of? What is your ideal partner like? What about your ideal relationship? (…)

Girl 1: The cool guy, no, that’s it, that’s more than clear. The typical bad boy no, because we don’t want a toxic person.

Girl 2: Well, someone who is a good person, kind …

Girl 1: And also egalitarian … respectful (…)

Researcher: Do you think that the intervention has helped you change that idea or that dream of …

Girl 1: Yes (…) maybe girls like the bad boy I don’t know why but … looking at it in another way, you stop liking him because of all the things you’ve seen and what can happen, then it’s like different and it changes your way of thinking and seeing that person (FG school 2)

In addition, some girls also gained self-confidence when they aligned their thoughts and preferences with the evidence shared with them. Some of them reported that what they learned in the interventions matched with their previous ideas about these issues. It reinforced their opinion preventing them to fall into violent relationships: “Girl 1: It is what we have said before, I already had my opinion formed and such, but it is true that with the project you can talk about it, and your ideas are more firm” (FG school 3). In some cases, they realized that having healthy or toxic relationships is a matter of choice. They can choose the type of relationship they want to have and make decisions and take steps to move away from unwanted situations, empowering them and enhancing their capacity of agency:

Girl 1: Yes (…) that helps us to know what we want too and if there is a person who is not for us, then we already have an idea of how to get away or how … or situations of how to say no, perhaps. (FG school 2)

## 4. Discussion

Our study has added evidence to the already available knowledge that identifies the peer group context as a socialization place for adolescents that shapes attitudes, preferences, and relationships [11,12,13,14,15]. In particular, our results suggest that preferences for non-egalitarian and potentially violent masculinities or egalitarian and non-violent masculinities in the group can influence the probability of girls being exposed to toxic intimate relationships. The information mentioned above is relevant knowledge to understand and tackle the public health issue of violence against women and girls. If attraction towards potentially violent masculinities and unattraction towards non-violent men and boys is related to a higher risk for girls to engage in toxic relationships, such as those characterized by group pressure and the mirage of upward mobility, it is of utmost importance to identify venues in which these preferences can be transformed, to prevent harmful relationships and promote healthier ones.

In our research, we shared with the adolescents the existing knowledge on the ill effects of toxic relationships in general, and gender violence in particular, on physical and psychological health [2,4,12,13], and the evidence of the different roles of different models of masculinity in reproducing or preventing gender violence [24]. We used this evidence as an instrument to help them reflect on their thoughts, preferences, and discourses in the peer group. The results showed that the evidence shared and discussed with them had a transformative impact on both boys and girls, which is relevant as overcoming gender violence is not a concern of only women or men, but of women and men together [32]. On the one hand, as a consequence of the evidence presented, girls acknowledged a change in their preferences. They went from choosing non-egalitarian and potentially violent boys to egalitarian and non-violent boys. To reaffirm their attraction towards non-violent boys indicates that this evidence had a protective effect, as it reduced the risk of girls being involved in toxic relationships such as those that occur as a consequence of peer group pressure or the mirage of upward mobility, which are characterized by a coercive discourse, and therefore tend to be associated with violent masculinities [33]. On the other hand, the fact that non-violent boys gained self-confidence in their non-violent attitudes indicates that the evidence-based interventions can prevent them from falling into more traditional and dominant masculinity behaviors due to group pressure on them, and therefore contribute to creating a group climate more conducive of healthy relationships. Therefore, the results support our hypothesis that knowing the negative impact that violent masculinities and toxic relationships have on health can help adolescents make informed decisions and transform their attraction to different types of masculinity, preventing toxic relationships to occur and creating a potential positive long-life impact on their health. These results show the importance of sharing and discussing scientific evidence regarding gender violence, its causes, and its consequences with adolescents, to prevent this reality among youth. It has implications for interventions from both the areas of health and education, which could take into account the transformative capacity of evidence, when it is accessible for youth, to achieve social impact. Our results indicate that adolescents’ attitudes and preferences that may lead to gender violence and potentially harmful effects on health can be transformed. Creating spaces of evidence-based dialogue where scientific evidence is presented and discussed is an effective strategy.

This study is not exempt from limitations. First, the study is based on self-reported data from the adolescents, and not directly observable data by the researchers, which is a limitation related to the nature of the object of study. In this regard, the interactions questionnaire collected adolescents’ knowledge of cases of peer group pressure or mirage of upward mobility, but did not provide direct evidence of their occurrence. As we do not have access to observe this reality, the questionnaire gave us a valuable indicator of its presence in the peer group. Second, we could not gather evidence of attitude transformation (or not) in boys reflecting a dominant traditional model of masculinity, which would be essential to reinforce the evidence of the transformative impact of the interventions. The focus groups as the primary instrument chosen to evaluate the potential impact on youths may have discouraged these boys from sharing their reflections in an open discussion. However, we identified changes in the peer group, in both boys and girls, which can be conducive to changes in these boys. Third, our research does not allow us to ascertain whether the interventions had an impact on the relationships youth eventually had or on their health status. Finally, the results of our study are not representative, as our aim was to conduct a descriptive analysis of the reality studied from an exploratory approach, which could be further analyzed in subsequent research. Further research could also focus on analyzing whether the impacts are extended to boys following the dominant traditional model of masculinity and evaluate the sustainability of the impacts on both boys and girls and their consequences in their physical and psychological wellbeing in the long term.

## 5. Conclusions

This study provided new evidence on how adolescents’ attraction to masculinities following a dominant traditional—and potentially violent—model can lead to the existence of toxic intimate relationships in the peer group, and how this reality can be transformed when youth gain knowledge about the damaging and pervasive consequences of violence on physical and psychological health. 

When adolescents know of cases of peer group pressure on girls to start a relationship, or cases of the mirage of upward mobility, which are examples of toxic relationships for girls, non-egalitarian and potentially violent boys tend to be more accepted and are seen as more attractive than when adolescents in the peer group do not know of these cases. Conversely, egalitarian non-violent boys tend to be more accepted and perceived as more attractive when the group does not know of such cases of toxic relationships than when they do know of them. This shows, on the one hand, that these types of relationships, which can have negative consequences on girls’ health, occur in the adolescents’ peer groups and, on the other hand, suggests that showing acceptance and attraction to boys representing a non-egalitarian and potentially violent model of masculinity may be a risk factor for girls to be exposed to toxic relationships, while acceptation and attraction of masculinity models showing egalitarian and non-violent values could be a protective factor. 

Results showed that the association between attractiveness and violence could be counteracted with scientific evidence about the impact on the health of violent relationships and the role of the different models of masculinity in promoting or preventing violence in intimate relationships. When this evidence was shared with adolescents, both boys and girls acknowledged the impact of this evidence on them. Non-violent boys started to feel more confident in their non-violent attitudes, reinforced them in the group, and gained arguments to respond to peer group pressure to adopt behaviors typical of the dominant traditional masculinities. In the case of girls, those who used to see those boys reflecting the dominant traditional masculinity as attractive started to dislike them and see egalitarian and non-violent boys as attractive, while those who already preferred egalitarian and non-violent boys reinforced their opinion. These changes create a safer climate in the peer group, where toxic relationships are more difficult to propagate, and healthier relationships are promoted.

## Figures and Tables

**Table 1:** Categories of analysis.

Categories	Description
1. Impact of the evidence of the negative consequences of toxic relationships and the role of masculinities	This category includes adolescents’ explanations of the perceived impact that the intervention sessions on health and masculinities had on them compared to the other interventions.
2. Consequences on boys: non-violent boys gain self-confidence in the peer group	This category includes boys’ explanations of the intervention program’s consequences (particularly sessions on health and masculinities) had on them.
3. Consequences on girls: girls redirected their attraction to non-violent boys	This category includes girls’ explanations of the intervention program’s consequences (particularly sessions on health and masculinities) had on them.

**Table 2:** Peer group talk about types of masculinity and peer group pressure on girls.

		28. Has a Girl in Your Group of Friends at the High School Ever Started a Relationship Because of What Her Friends Told Her?			
		28. (a) Yes		28. (b) No	
		*N* ^4^	%	*N*	%
20. How do your group of friends at the high school speak about non-egalitarian boys, “bad boys”? [DTM] (select a maximum of 5 options)	Group talk showing acceptance and attraction ^1^	187	66%	98	63%
	Group talk showing rejection and unattraction ^2^	46	16%	41	26%
	Other responses ^3^	51	18%	17	11%
	Total responses	284	100%	156	100%
22. How do your group of friends at the high school speak about egalitarian boys who are not self-confident? [OTM] (select a maximum of 5 options)	Group talk showing acceptance and attraction	111	44%	93	54%
	Group talk showing rejection and unattraction	107	42%	54	31%
	Other responses	36	14%	26	15%
	Total responses	254	100%	173	100%
24. How do your group of friends at the high school speak about egalitarian and self-confident boys? [NAM] (select a maximum of 5 options)	Group talk showing acceptance and attraction	199	76%	151	78%
	Group talk showing rejection and unattraction	24	9%	11	6%
	Other responses	39	16%	31	16%
	Total responses	262	100%	193	100%

^1^ Participants selected one or more of the following responses: “They are guys who are talked about as friends, or who are treated as friends”; “They are guys who are attractive, who are liked”; “They are guys who are talked about often”; “They are guys that the majority finds them interesting”; “They are guys with whom the majority like to talk”; “They are popular boys”; “Sometimes it is said that these boys are liked a lot among the girls”. ^2^ Participants selected one or more of the following responses: “They are guys who are never or hardly ever talked about”; “They are guys who are sometimes laughed at”; “They are guys that the majority do not like”; “Sometimes it is said that these boys are not liked a lot among the girls”. ^3^ Other responses that are not taken into account for the present study were: “It is said that they are the ideal boys for a dating relationship”; “It is said that they are the ideal guys for a hook up, a one-night stand or a few days, not boyfriends”; “These issues are not discussed”; “Other response” (open question). ^4^ Frequencies and percentages are based on the number of responses and not on the number of participants, as each participant could select more than one response in questions 20, 22, and 24.

**Table 3:** Peer group talk about types of masculinity and mirage of upward mobility on girls.

		25. Girls Feel Attracted to Boys with Attitudes of Disrespect towards Them [Mirage of Upward Mobility]. Do You Know Any Situation of This Kind?			
		25. (a) Yes		25. (b) No	
		*N* ^4^	%	*N*	%
20. How do your group of friends at the high school speak about non-egalitarian boys, “bad boys”? [DTM] (select a maximum of 5 options)	Group talk showing acceptance and attraction ^1^	210	67%	71	55%
	Group talk showing rejection and unattraction ^2^	48	15%	39	30%
	Other responses ^3^	54	17%	18	14%
	Total responses	312	100%	128	100%
22. How do your group of friends at the high school speak about egalitarian boys who are not self-confident? [OTM] (select a maximum of 5 options)	Group talk showing acceptance and attraction	115	41%	88	58%
	Group talk showing rejection and unattraction	120	43%	38	25%
	Other responses	44	16%	26	17%
	Total responses	279	100%	152	100%
24. How do your group of friends at the high school speak about egalitarian and self-confident boys? [NAM] (select a maximum of 5 options)	Group talk showing acceptance and attraction	182	69%	142	73%
	Group talk showing rejection and unattraction	35	13%	22	11%
	Other responses	47	18%	31	16%
	Total responses	264	100%	195	100%

^1^ Participants selected one or more of the same responses as in Table 2. ^2^ Participants selected one or more of the same responses as in Table 2. ^3^ Other responses were the same as in Table 2. ^4^ Frequencies and percentages are based on the number of responses and not on the number of participants, as each participant could select more than one response in questions 20, 22, and 24.

**Table 4:** The average score of the utility perceived by adolescents in the different interventions.

Intervention	Average Score ^1^
Intervention 1	3.09
Intervention 2	2.47
Intervention 3	3.06
Intervention 4	3.30
Intervention 5	3.31
Intervention 6	3.04
Intervention 7	2.93
All interventions	3.06

^1^ Scores range from 1 to 5 (1. Not useful at all; 2. A little useful; 3. Quite useful; 4. Very useful; and 5. Totally useful).

## Data Availability

The data presented in this study are available on request from the corresponding author.
